# Terahertz-range on-chip local oscillator based on Josephson junction arrays for superconducting quantum-limited receivers

**DOI:** 10.3762/bjnano.16.158

**Published:** 2025-12-22

**Authors:** Fedor V Khan, Lyudmila V Filippenko, Andrey B Ermakov, Mikhail Yu Fominsky, Valery P Koshelets

**Affiliations:** 1 Kotel’nikov Institute of Radio Engineering and Electronics of RAS, 11/7 Mokhovaya st., Moscow, 125009, Russian Federation; 2 Moscow Institute of Physics and Technology, 9 Institutskiy per., Dolgoprudny, Moscow Region, 141701, Russian Federation

**Keywords:** Josephson junction arrays, phase-locking, superconducting local oscillator, superconductor integrated receiver, terahertz-range oscillators

## Abstract

In this paper we present the results of the development, fabrication, measurements, and analysis of terahertz-range oscillators based on Josephson junction arrays embedded into the central electrode of a coplanar line. The influence of array geometry, the presence of a matched load at the nonradiating edge, and the magnitude of the tunneling current density of Josephson junctions on such oscillator characteristics as radiation power, linewidth, and operating range are discussed. Various options are suggested for further improvement of the oscillator performance.

## Introduction

Superconducting heterodyne receivers based on superconductor–insulator–superconductor (SIS) tunnel junctions have ultimate characteristics unreachable to devices based on other principles [1–3]. The unique nonlinearity of the current–voltage characteristic (IVC) near the gap voltage of the junction enables the gain of the intermediate frequency (IF) signal after mixing with the radiation from the local oscillator (LO) [4]. This effect together with the operation at cryogenic temperatures and the quantum nature of the mixer itself allow for the development of receivers with the noise temperature *T*_n_ only a few times higher than the quantum limit ≈*hf*/*k*_B_, where *h* and *k*_B_ are the Planck and Boltzmann constants, respectively, and f is the frequency of the incoming radiofrequency (RF) signal [4–5]. Such receivers are used as a sensitive element of the state-of-the-art terahertz (THz) range ground-based (ALMA [6], APEX [7]) and space-based (Hershel [8], Millimetron [9]) telescopes. The ground and space-based telescopes are combined in a large net called “Event Horizon Telescope” [10] which was used for direct observation of the black hole shadow in the M87 Galaxy [11].

The LOs for the conventional SIS receivers are mostly generated by Schottky diode multipliers, which have a radiation power in the terahertz range up to 2 mW and efficiency of approx. 5% [12]. However, these devices can be fabricated only in few laboratories around the world (e.g., JPL NASA) and may not be accessible in some countries.

The other approach was suggested in previous works [13–14] where the LO based on the FFO is fabricated on the same chip and in the same technological process as that of the SIS mixer. This approach allowed for superconductor integrated receivers (SIRs) with some of the characteristics even superior to those of SIS receivers with conventional LO based on Schottky diodes. The concept of the SIR has proven itself for many times both in laboratory and in real-life conditions. The terahertz SIS-mixer integrated on the same chip with the LO based on a flux-flow oscillator (FFO) and a receiving antenna [13] was used to study the irradiation of the human body [15], gas spectroscopy [16], and the atmosphere gas components at the TELIS mission on board of the stratospheric balloon [17].

However, the LO based on FFO has some problems yet to be solved. The operation frequency of the FFO is limited by the gap frequency of the superconducting niobium that forms one or both of its electrodes [18]. Currently, Josephson junctions (JJ) are mostly fabricated using Nb–Al/AlO*_x_*–Nb or Nb–Al/AlN–NbN technologies [19–20]. A number of attempts were made to establish the technology for the fabrication of Nb-based technologies, such as NbTiN–Al/AlN–NbTiN [21], NbN–AlN–NbN [22], NbN–TaN–NbN [23], NbN–MgO–NbN [24]. Nontheless, the use of Nb alloys will only partially solve the problem of high-frequency generation due to high surface losses in NbN and NbTiN [18,25–26]. Note that the use of Nb–Al/AlN–NbN allows for the fabrication of tunnel junctions with a tunnel current density of up to 100 kA/cm^2^ with a quality ratio *R*_j_/*R*_n_ over 20, where *R*_j_ and *R*_n_ are the resistance of the SIS junction below and above the gap voltage *V*_g_, respectively [20]. A high tunnel current density is crucial for the RF and IF bandwidth of the mixer; however, to the best of our knowledge, the FFO based on Nb alloys has not been developed yet.

Another problem is related to the fact that the FFO operates in resonant (or Fiske-steps) mode [27] at voltages below *V*_g_/3. At higher voltages, the presence of the self-pumping effect [28] leads to an increase of the attenuation of the waves propagating in the FFO and the smoothing of the Fiske steps. The operation in the Fiske steps mode is complicated by the need to search for operation points at a particular frequency with sufficient generation power. The computer procedure of the operating point selection performed by varying the bias current through the FFO and the magnetic field takes about 1 min, and it is done after each thermal cycling [13]. Also, the ratio of the radiated power to the consumed DC power is slightly higher than 5%.

Some of the aforementioned problems can be solved by the use of Josephson junction arrays as LO for the SIR. The junctions can be fabricated by the use of robust Nb-based technologies, whereas the electrodes of the transmission lines where the junctions are implemented can be fabricated using Nb compounds. In a work by Uzawa et al. the generation of up to 800 GHz was observed [29]. When a JJ array is properly matched to the load, its radiation power linearly increases with the number of synchronized junctions *N*, while the emission linewidth linearly decreases [30–31]. Moreover, if reflections from the ends of the array are suppressed (e.g., by using a matched load), standing waves and resonances should no longer occur. It should be noted that if the characteristic synchronization radius of the junctions in the array is smaller than the length of the array, there is a saturation of the maximum power that can be achieved [32].

Currently, there are several implementations for the arrangement of the JJs in the array. The junctions can be connected either in series and or in parallel and can be arranged in several ways: spaced by λ/2 from each other, at a distance much smaller than λ, or arranged in groups, where junctions within each group are closely packed while the groups themselves are separated by λ/2 [31,33]. Here, λ is the wavelength at the frequency of operation. When the junctions are grouped or spaced by λ/2, mutual synchronization becomes easier to achieve. However, in such cases, the generation spectrum is limited to only a few frequencies determined by the condition that the spacing between the groups or individual junctions contains an integer number of half-wavelengths. One-dimensional and two-dimensional array circuits have also been implemented. In the work [34], with 1986 JJs arranged in a two-dimensional array at λ/2, a power of 160 μW was achieved at a frequency of 240 GHz. In the paper [35], a one-dimensional array consisting of 9996 Nb/Si/Nb junctions was presented, capable of frequency tuning from 139 to 343 GHz, with a linewidth in the best points of less than 100 kHz. In the works by Uzawa et al. [29,36–37], generators and receivers based on one-dimensional arrays, where the JJs are spaced at λ/2, were investigated, demonstrating the possibility of generating frequencies from 150 to 800 GHz with power ranging from 0.2 to 10 μW.

In recent papers [38–39], we have suggested a new type of JJ arrays where the junctions are embedded into the central electrode of the coplanar transmission line (CPW). For a local oscillator in a heterodyne receiver, continuous frequency tuning is crucial. For this reason, the junctions are spaced at distances much smaller than the wavelength. We suppose that the choice of such topology where the array is in the central electrode of the CPW enabled the synchronization between the junctions by high frequency currents. This allowed to achieve the power of up to 0.4 μW for the array consisting of 200 junctions with an area of 2.8 μm^2^ and a tunnel current density of approx. 5 kA/cm^2^. This power is already sufficient for on-chip applications: both for the mixer pump in the receiver and the operation of the harmonic mixer (HM) for frequency and phase locking. For the first time the possibility of phase locking of the JJ array to the local source unit was demonstrated with a spectral ratio higher than 90% at the best points [39]. We have also studied and compared the free-running linewidth of the array to theoretical estimates [32,40–41] and investigated how the array performance is affected by the matched load in the form of Klopfenstein tapering, which ends with the section of the microstrip line with the normal top electrode.

In this paper we concentrated on the high-frequency properties of JJ arrays and on the discussion about the feasibility of the LO in SIR based on them. In our previous works we observed that the radiation power at frequencies higher than 500 GHz was lower than expected. However, for the samples with higher tunnel current density, the operation frequency was observed to be higher. Here, we will give the explanation and suggest the methods for further increase of the maximum operating frequency of the JJ arrays. In addition, we fabricated a new set of samples that incorporate a matched load on the non-radiating end, improved array–SIS detector matching circuits, and varied tunnel-current densities.

The LO for SIR should satisfy the following criteria based on the previous study of a heterodyne receiver with a local oscillator based on long Josephson junctions (LJJ) [42]. First, the power of the RF signal incoming to the SIS mixer should induce the quasiparticle current step more than 0.25 of the current at the gap voltage of the mixer junction. Second, the oscillator frequency should be tunable in a wide range. Third, the ability to phase-lock to the external stable synthesizer should be implemented. Fourth, the spectral ratio (ratio of the signal power at the peak to the total radiated power, including noise and parasitic modes) in the PLL mode should exceed 90%. In this paper we will show that newly fabricated samples meet these criteria.

The paper is divided into four sections. In the first section we briefly review the topology and the fabrication technology of the samples. In the second we describe the measurement setup and methodology. After that we present the results of the measurements with the calculations and simulation. Finally, we discuss the feasibility of the LO based on JJ arrays of the proposed topology.

## Experimental

### Samples

**Figure 1:**
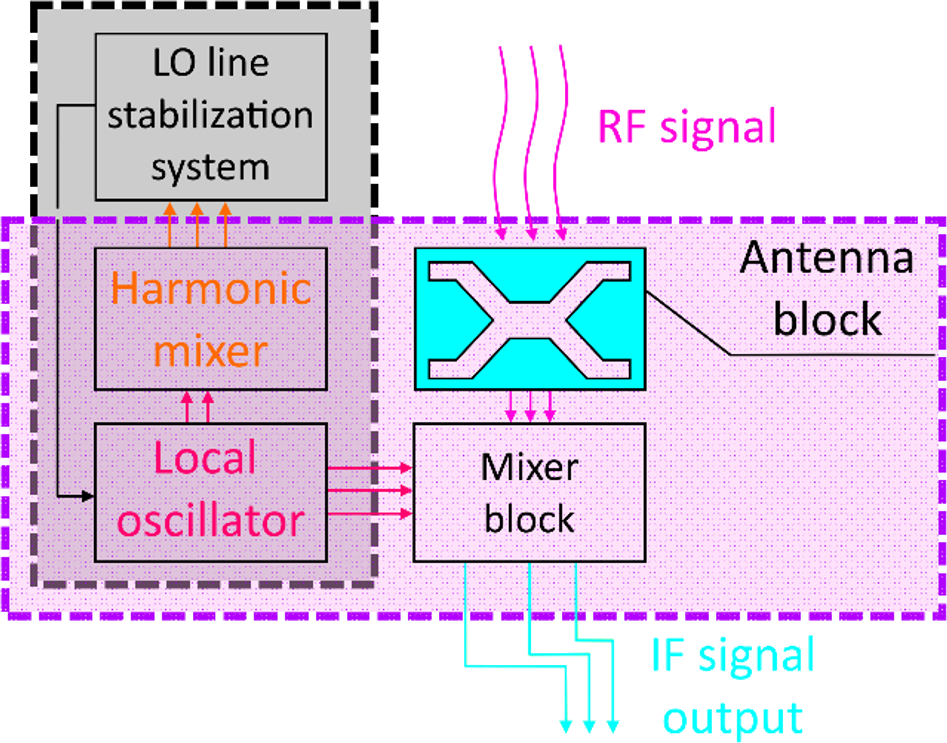
Principal scheme of the superconductor integrated receiver (SIR).

The block diagram of the SIR is shown in Figure 1. The incoming RF signal is fed into the antenna and then goes into the mixer, where it is mixed with the signal from the LO. Due to the extreme nonlinearity of the SIS mixer, the signal is down-converted to the IF with moderate loss [43]. In order to stabilize the generation line of the LO, the system with the HM is used. HM is a single Josephson tunnel junction that not only helps to estimate the array radiation power while operating as a direct detector, but also mixes the radiation from the array with the high-order (up to 40) harmonics of the external commercial synthesizer (operation frequency range: 16–19 GHz). The IF signal resulting from the HM goes to the LO stabilization system, where its frequency is compared with the reference and the bias current through the array is adjusted in order to minimize the difference between the IF signal and the reference. The details on the operation principles of this system can be found in [13]. All the elements within the magenta rectangle in Figure 1 are integrated in a single chip of the SIR.

Here we study the local oscillator based on the JJ array together with the harmonic mixer (elements within the black dashed rectangle in Figure 1). The purpose of the samples under study is to develop the wideband matching circuits between the LO and HM and implement the phase locking loop (PLL) for a new type of LO [42].

**Figure 2:**
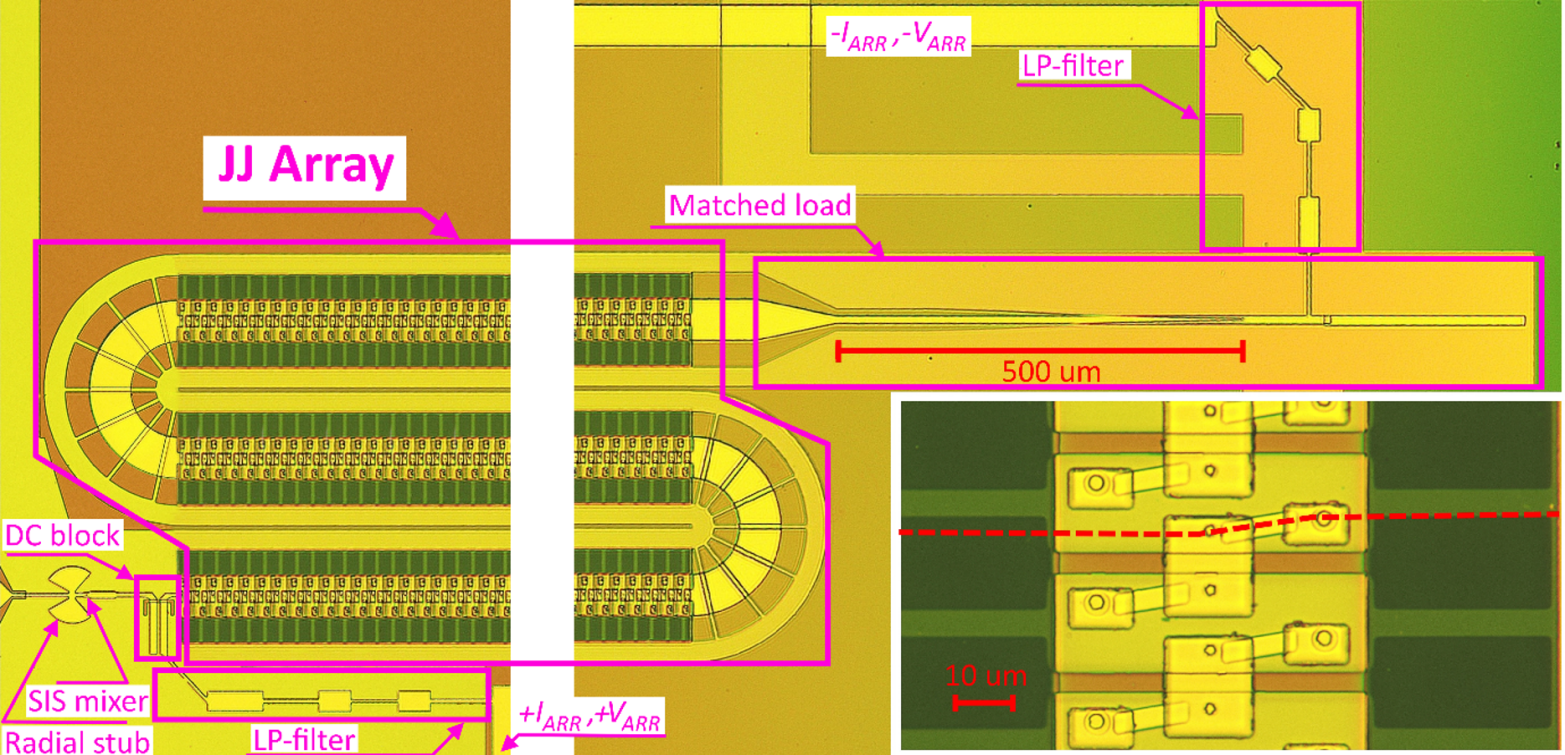
Capture of the experimental sample by an optical microscope; inset – enlarged image of the junctions in the array. The length of each of the three sections of the array is 2 mm (the major part is not shown for visibility). The dashed red line shows the cross-section of the layers illustrated in Figure 3.

The image of one of the experimental samples captured by an optical microscope is shown in Figure 2. The radiation from the array of the Josephson junctions is detected by a small SIS tunnel junction with an area of approx. 1 μm^2^. This junction also functions as the harmonic mixer in spectral measurements. A DC block in the form of a slot antenna allows to separate the DC connection of the array and the detector. The SIS tunnel junction has a parasitic capacitance of approx. 100 fF that becomes significant at terahertz frequencies. The microstrip line of a certain length shorted at high frequencies by a radial stub serves as the effective inductance in order to compensate the parasitic capacitance in the band of operation. The connection of the JJ array to the DC bias supply is implemented using Chebyshev low-pass filters (LP filter in Figure 2) in order to prevent the array radiation from leaking into these lines.

In order to suppress the standing waves in the array, the matched load in the form of Klopfenstein tapering [44] is used. It is made up by a 5λ/4-long transition from CPW to the microstrip line with a normal top electrode that serves for attenuation of the reflected wave.

Each JJ in the array is shunted by a thin-film resistor made of normal metal. In our technological cycle, the molybdenum film with a thickness of 100 nm (surface resistance of approx. 1 Ω/square) is used. A shunt is crucial for reducing the McCumber parameter to the value of ≈0.3 in order to provide a hysteresis-free current–voltage characteristic (IVC) [3]. The parameters of the single junctions in this work are as follows: *I*_c_ = 110 μA, *C* = 300 fF, *L*_shunt_ = 1.5 pH, and *R*_shunt_ = 1.9 Ω. For this set of parameters there is a bump on the single junction IVC at a voltage of approx. 0.5 mV caused by an LC resonance between the shunt inductance and the junction capacitance. Since the corresponding resonance frequency is below the operating range of the samples, we will not discuss these effects here. At certain parameter values, the IVC of the single JJ exhibits a number of features that can be quantitatively described by the RLCSJ model, as shown in [38,45–46].

**Figure 3:**
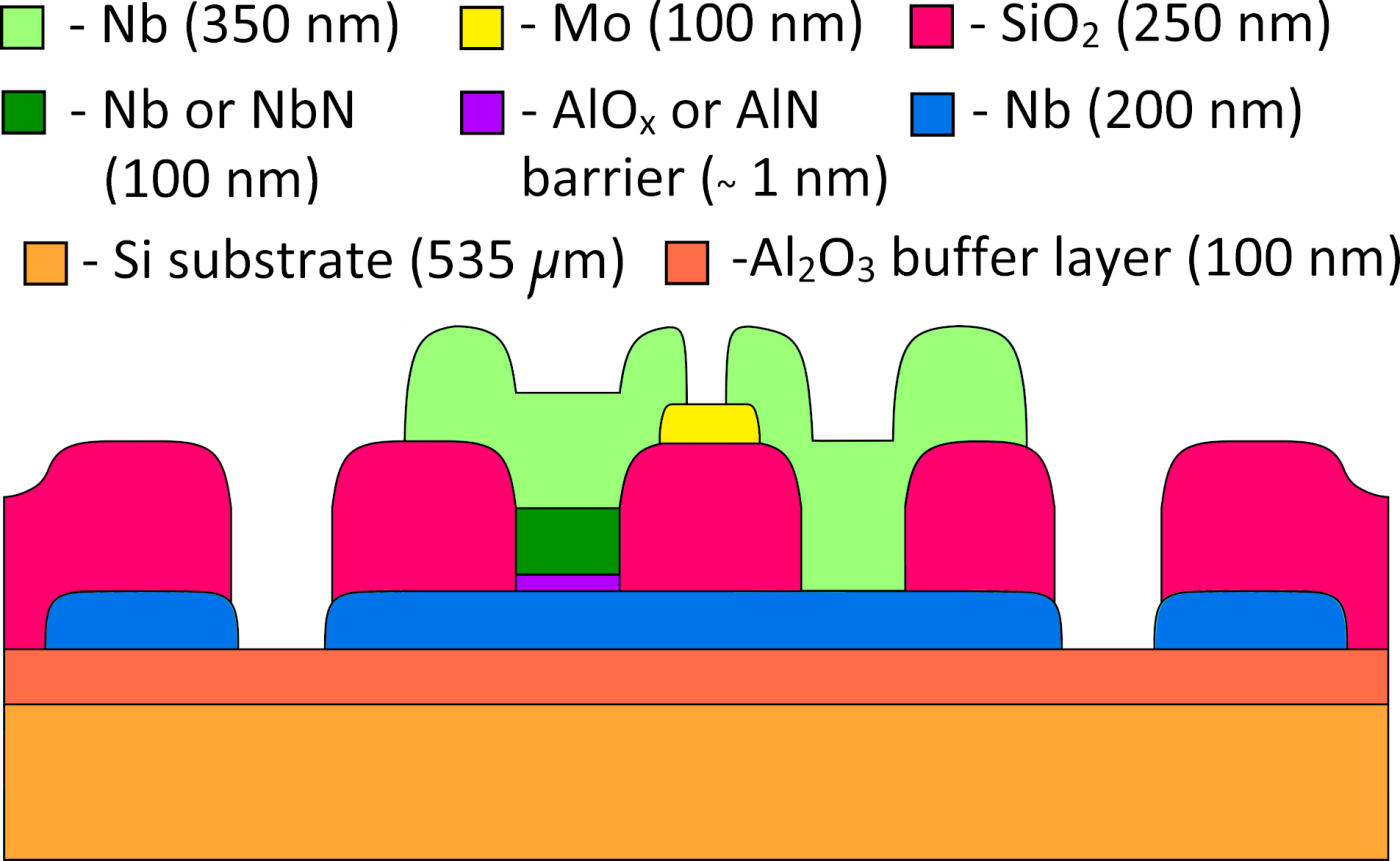
Cross-section of the technological layers near the JJ and the shunt (see the red dashed lines in Figure 2 and the inset).

The image of the technological layers near the JJ and the shunt in the cross-section shown by the dashed line in Figure 2 is shown in Figure 3. The details of the fabrication processes can be found in our previous works [19–20].

### Measurement technique

When the JJ array irradiates the SIS mixer, the probability of quasiparticle tunneling through the tunnel barrier increases. This leads to a rise in current at voltages below the gap and the emergence of the so-called quasiparticle steps. This process is known as photon-assisted tunneling. The magnitude of the current on a quasiparticle step within the investigated range is linearly proportional to the incident power. The precise shape of the pumped IVC can be derived from the known autonomous IVC using the expressions from the Tucker–Feldman model [47]. The model is applicable for all frequencies and powers in the study, provided that a sufficient number of harmonics *p* are taken into account when calculating the pumped IVCs from an autonomous IVC. We selected *p* = 5, since beginning from *p* = 3 the change in the form of the calculated IVCs was comparable to the approximation error. However, one should take into account that a high incident power would likely cause junction overheating and give rise to nonequilibrium effects, thus restricting the model applicability [48].

**Figure 4:**
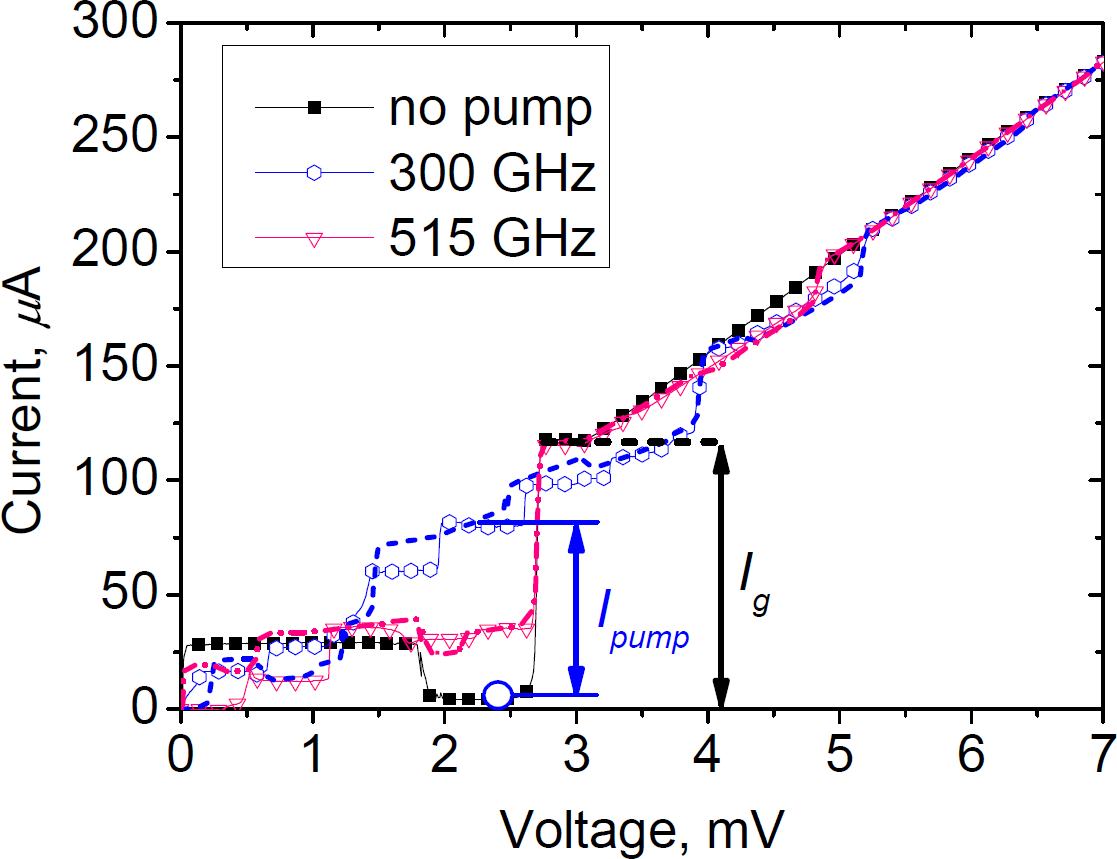
The set of the IVCs of the SIS mixer irradiated by the JJ array with 600 JJs at frequencies of 300 GHz and 515 GHz. The dashed curves are calculated using theoretical expressions from Tucker–Feldman model [47]. Circle at 2.4 mV denotes the operating point of the SIS mixer during DC measurements. *I*_pump_ is the current through the mixer that arises from photon assisted tunneling and serves for the incoming power estimate. The power level is about 0.18 μW at 300 GHz and 0.07 μW at 515 GHz. *I*_g_ is the current at the gap voltage *V*_g_.

A set of IVCs for the SIS mixer at various pump power levels is shown in Figure 4. Experimental IVCs are indicated by colored markers, while the dashed curves represent approximations obtained using the Tucker–Feldman expressions. Due to the incompletely suppressed critical current, Shapiro steps also appear on the IVC of the SIS mixer at voltages of *hf*/2*e* [49]. The corresponding jumps in the theoretical IVCs arise from replicas generated when shifting the autonomous IVC by *nhf*/*e*, where *n* is an integer.

**Figure 5:**
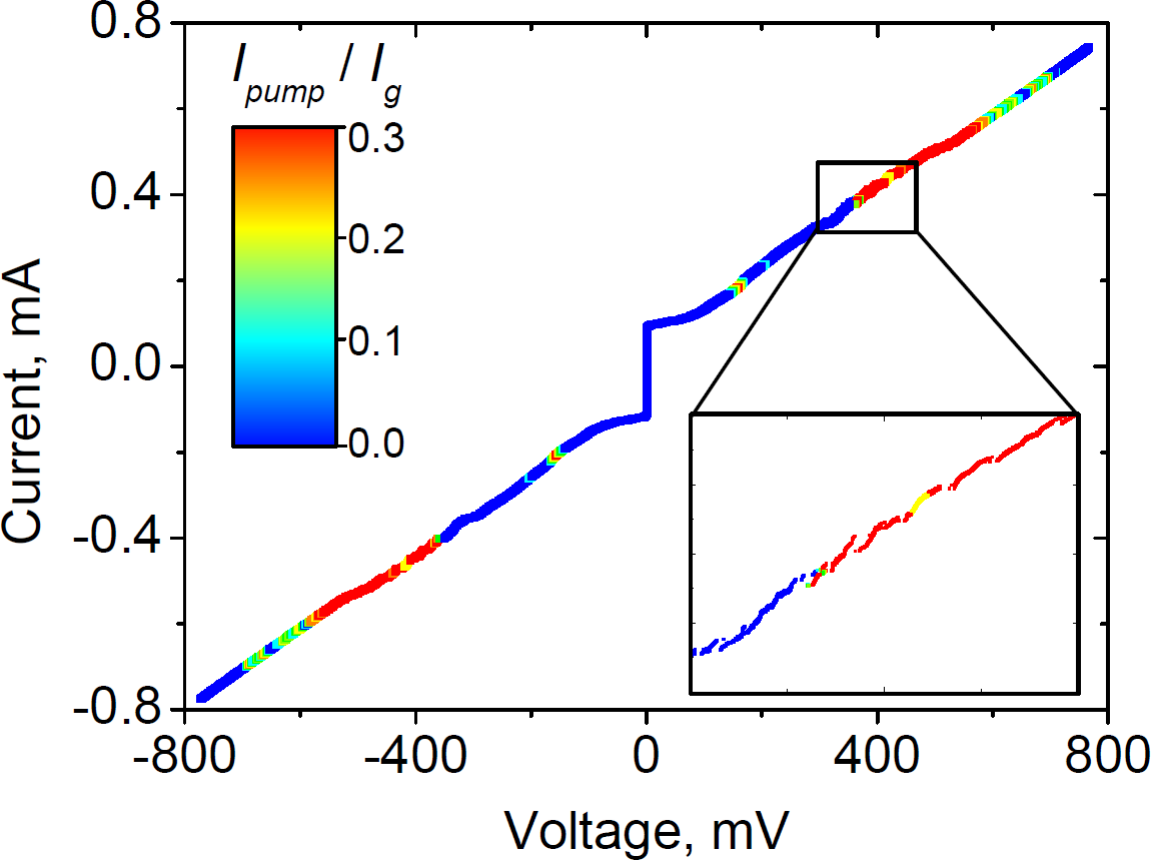
IVC of the 600 JJ array with 300–550 GHz matching circuit and matched load on the nonradiating end. The color indicates the *I*_pump_ of the SIS mixer in the corresponding operating point. The inset shows the enlarged part of the IVC, where the steps related to the resonances along the entire array length are observed.

Figure 5 shows the IVC of the JJ array for one of the samples with 600 JJs. The color indicates the value of the *I*_pump_ normalized to *I*_g_ for the SIS mixer at the corresponding voltage. The steps present on the IVC (see the inset in Figure 5) arise from longitudinal resonances along the entire length of the array, which are much smaller than in previous samples with no matched load (see [38] to compare). The relation between the voltage across the array and the radiation frequency can be estimated using the expression *f* = 2*eV*/*hN*, where *N* is the number of synchronized junctions in the array. This formula holds with good accuracy, but is only suitable for qualitative estimates. Moreover, previous measurements have demonstrated that the actual generation frequency may deviate from the value calculated by the above formula since the generation frequency locks on the modes of the coplanar waveguide, and due to the fact that not all of the junctions in the array are synchronized [50].

## Results and Discussion

### Operation frequency band of the samples

**Figure 6:**
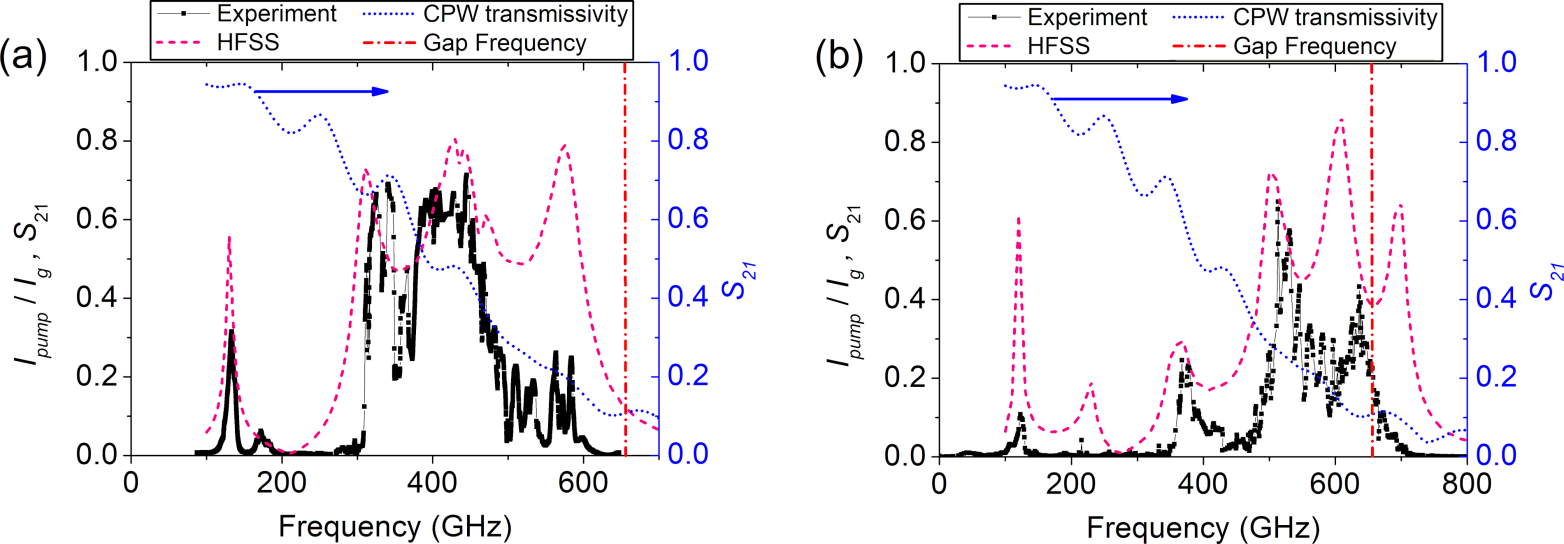
Pump current (black squares) and the calculated *S*_21_ parameter (dashed pink curve). (a) Design for the 300–550 GHz range and (b) 500–700 GHz range. The blue curve (right axis) denotes the transmissivity of the CPW line. The red vertical line depicts the gap frequency for the samples.

We performed numerical calculations in order to design the coupling circuits between the JJ array and the SIS mixer. The calculation methods for the superconductor planar structures are described in works [51–52] and will not be duplicated here. In total, two designs were calculated and tested, covering the 300–550 GHz and 500–700 GHz range. The first matching circuit has a bandwidth of more than 50% relative to the central frequency (250 GHz with a central frequency of 450 GHz); the second, ≈30% (bandwidth 200 GHz with a central frequency of 600 GHz). The low-frequency matching circuit was easier to design due to the dispersion in the superconductor microstrip lines made of niobium at frequencies over 500 GHz, rising from the frequency dependence of the London penetration depth [18,53]. The experimental results for the S_21_ coefficient of the 300–550 GHz and 500–700 GHz matching circuits and the calculations are shown in Figure 6a and Figure 6b, respectively. The experimental values of *I*_pump_ are normalized to *I*_g_. However, the experiment shows a discrepancy with the calculations at frequencies over 500 GHz (see Figure 6a).

We studied the possible reasons for this and found that the performance deterioration at high frequencies is likely caused by the attenuation in the CPW. We performed calculations of the CPW with the same material parameters as the those in the sample arrays. The *S*_21_ parameter for the coplanar line is shown by the cyan dotted line in Figure 6a and Figure 6b. The values are indicated on the right axis. The decrease is caused by the leak of the JJ array radiation into the substrate. Moreover, the parameters of the films that form the electrodes for this technological run turned out to be slightly different from those used in the calculations (in particular, the gap frequency, denoted by the vertical dash-dotted red line was found to be 650 GHz instead of 700 GHz). The estimate of the gap frequency was done from the value of *V*_gap_ of the tunnel junctions (see Figure 4, where *V*_gap_ ≈ 2.7 mV, which corresponds to the gap frequency of niobium films ≈650 GHz).

**Figure 7:**
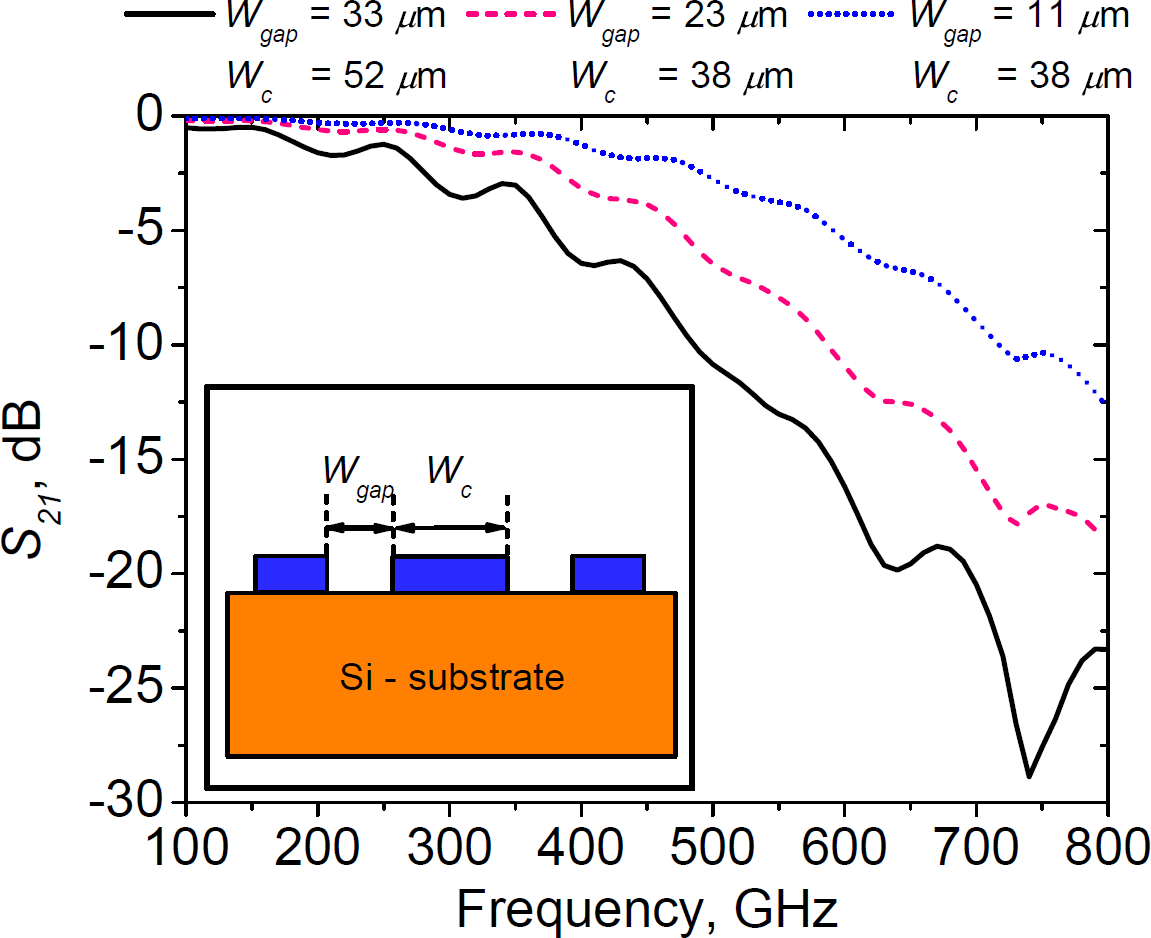
Transmissivity of the CPW lines with different gap widths. The inset shows the profile of the CPW.

A possible solution to address this problem is the CPW with a smaller gap width *W*_gap_ (see the inset in Figure 7). As can be seen from Figure 7, with a decreasing *W*_gap_ the attenuation in the CPW becomes smaller. This approach will be used in the topology of new designs. The width of the central electrode *W*_c_ is also changed in order to keep the characteristic impedance close to 50 Ω and the effective dielectric constant close to the ones in this study. However, the width of the central electrode of the CPW *W*_c_ = 38 μm is limited in the present design by the geometrical size of the junctions shunted by a thin-film resistor. As discussed in [54], for the CPW lines with a high *W*_c_ and small *W*_gap_, the difference between the frequencies of the odd and even modes becomes smaller. The coexistence of these modes is likely to degrade the oscillator performance because two modes with different wavelengths, and therefore different propagation constants, will be present in the CPW line, which may hinder phase-locking to the external stable synthesizer. In addition, a significant change in the characteristic impedance or propagation constant will likely affect the synchronization radius [32]. This topic will be addressed in future research.

**Figure 8:**
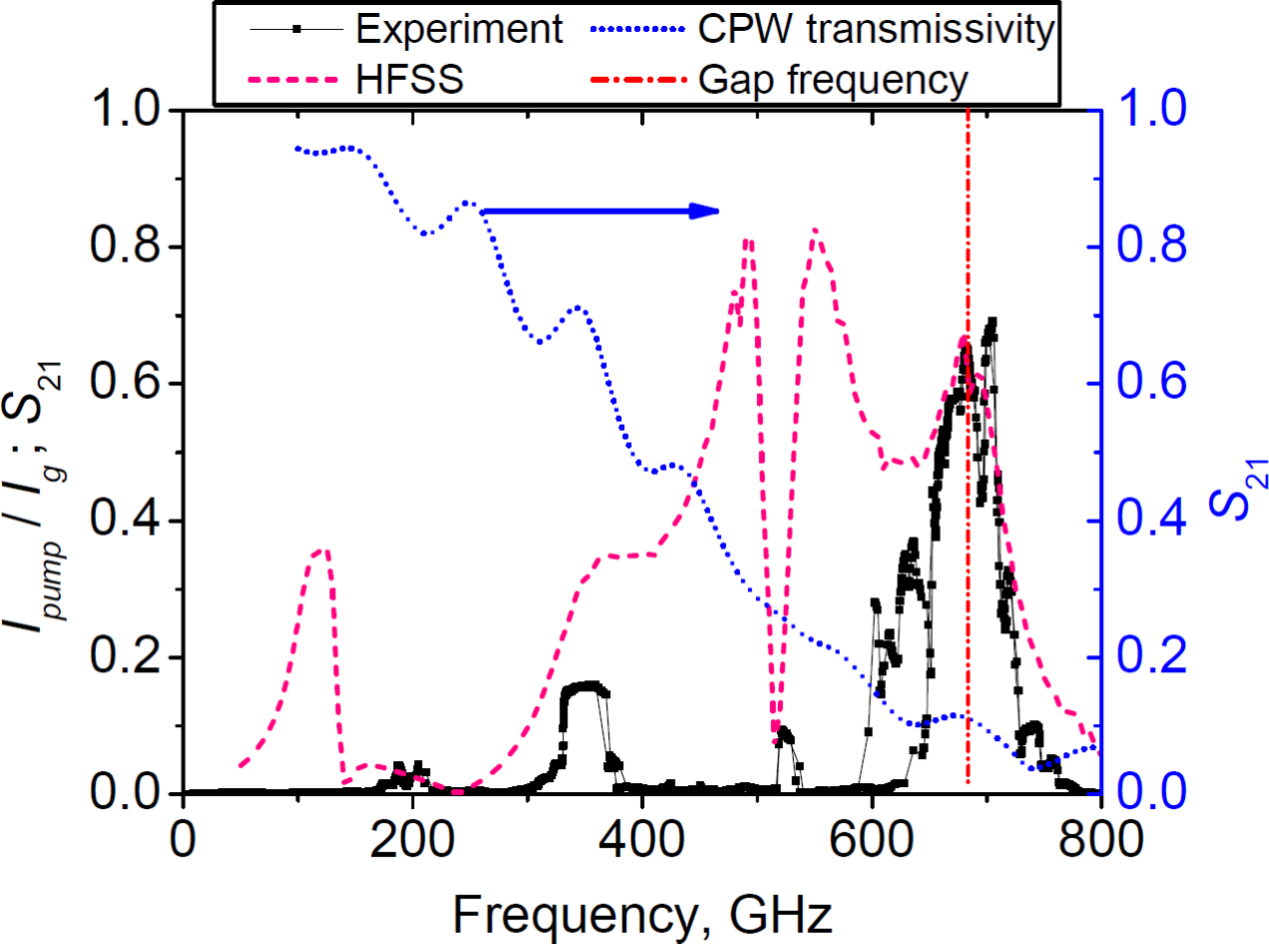
Pump current and calculated *S*_21_ parameter for the scheme with a higher critical current density. The discrepancy between the experiment and modeling is likely to be caused by the absence of the matched load at the nonradiating end.

We also compared the new results for the tunnel current density of 5 kA/cm^2^ to the previous series with a 13 kA/cm^2^ tunnel current density; the results are shown in Figure 8. It should be noted that the radiation power is higher for samples with a higher tunnel current density. Although synchronization and stable generation were not achieved across the entire bandwidth supported by the matching circuit, due to the absence of a matched load at the nonradiating end, the maximum operating frequency still was as high as 700 GHz. The possible reason for the better performance of the samples with a high tunnel current density at high frequencies is that with an increased tunnel current density, a bigger part of the RF current is flowing through the junction and less through the capacitance and the shunt (which also has finite inductance that deters high-frequency properties of the arrays). The new series of the experimental samples with higher tunnel current density is currently being fabricated.

### Spectral properties of the Josephson junctions array

**Figure 9:**
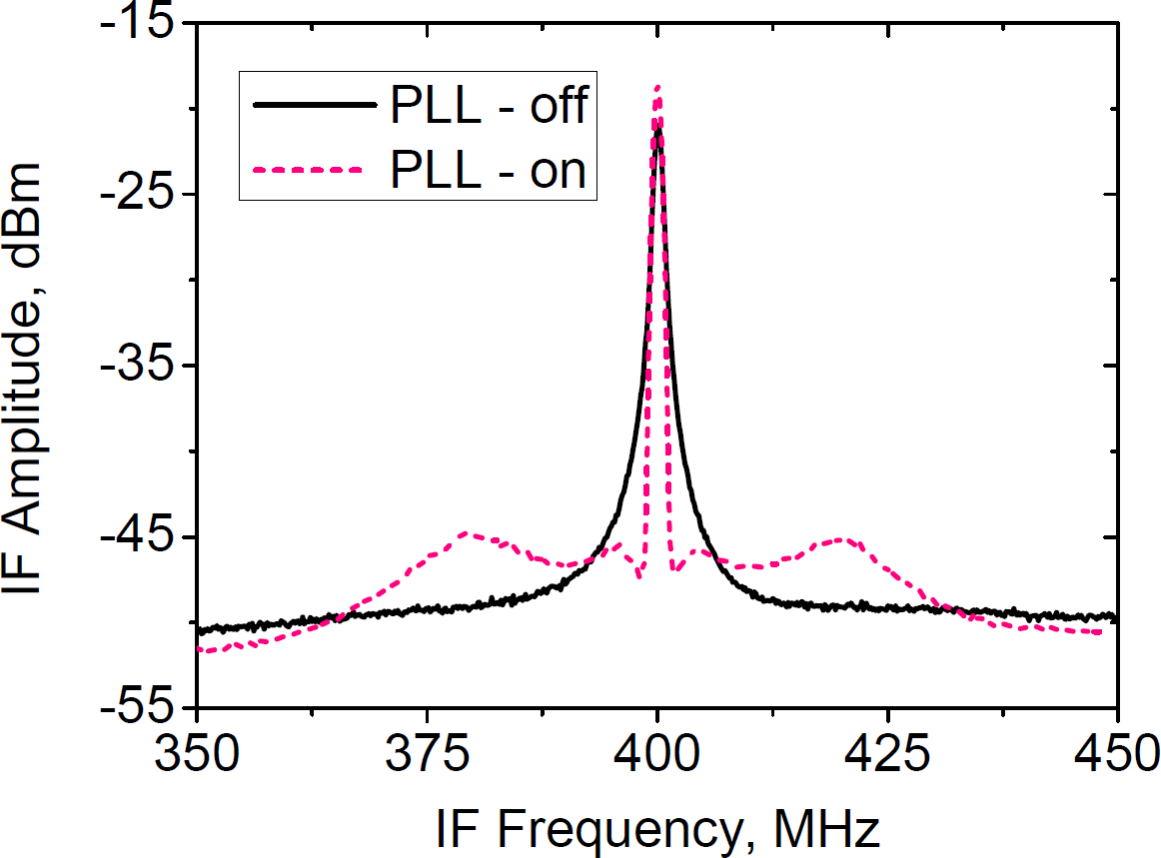
IF spectra of the 600 JJ arrays at 522 GHz. The free-running radiation linewidth is ≈0.5 MHz with a signal-to-noise ratio of ≈30 dB; the spectral ratio is 92% in case if the array operates in PLL mode.

In this section we will discuss the results of the spectral measurements and the feasibility of the LO in SIR based on a JJ array of the proposed topology. First, for the SIR to operate, the power of the LO coupled to the SIS junction induces the current on the quasiparticle step greater than 25% of the *I*_g_ [13]. The measurements from the previous section show that this requirement is confidently met. Second, the LO radiation must be sufficiently monochromatic (spectral ratio ≥90%) in order to prevent signal spectra change while down-conversion to IF. Furthermore, for applications in spectroscopy, phase-locking to the external stable synthesizer is crucial [16]. Figure 9 shows the radiation IF spectrum of the JJ array at a frequency of 522 GHz. The linewidth of the free-running array is less than 0.5 MHz, while the signal-to-noise ratio is approx. 30 dB. The calculation using the Rogovin–Scalapino [40–41] formula with the differential resistance normalized to a single junction yields the value of approx. 0.1 MHz. This is somewhat lower than the experimental value, probably due to the presence of the thermal noise arising from the current flowing through the shunts and other low-frequency fluctuations. In our earlier works we demonstrated the feasibility of phase-locking the array to an external stable synthesizer. The spectrum in the PLL mode is shown by the dashed pink line in Figure 9. The spectral ratio reaches 92%, which is sufficient for the integrated receiver operation. In the PLL mode, the linewidth becomes very small due to the elimination of the influence of both low-frequency and high-frequency noises; its measured value is determined by the resolution bandwidth of the spectrum analyzer. The detailed study of the phase noise in the PLL mode is subject for further research.

**Figure 10:**
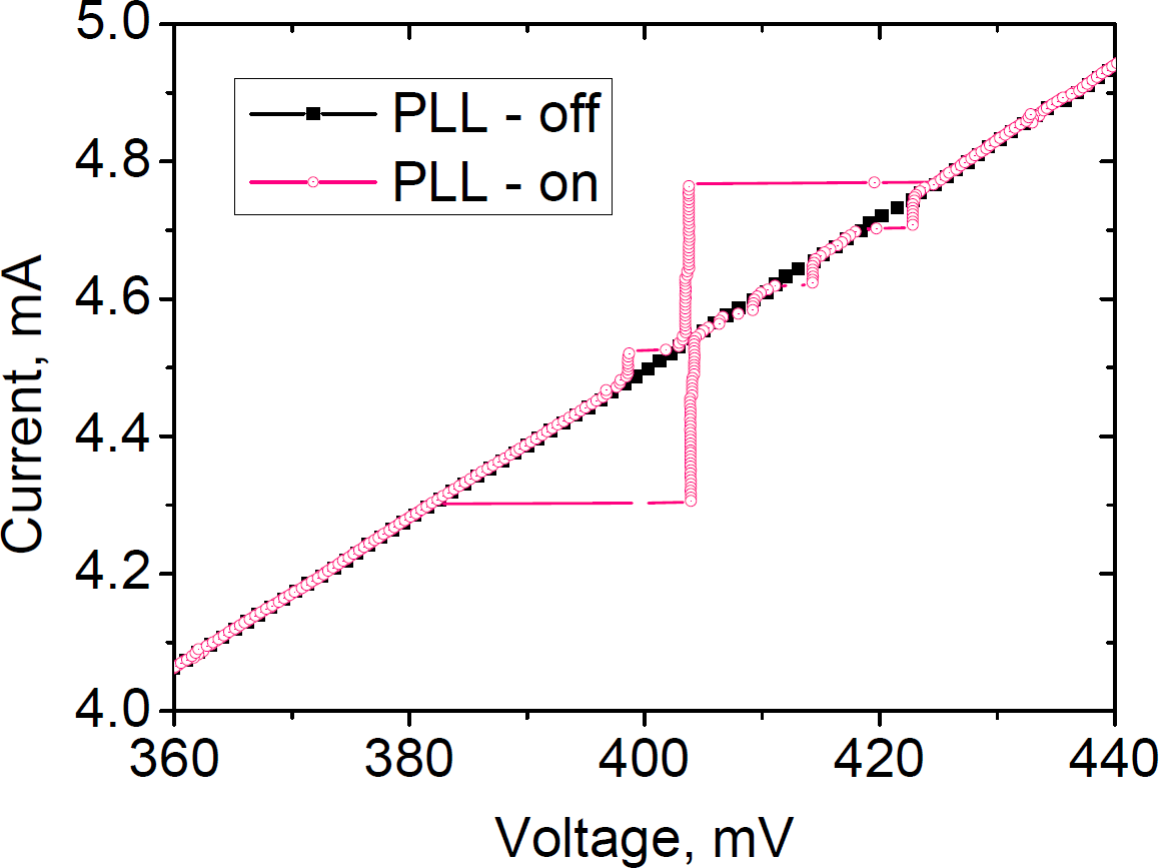
JJ array IVCs with (pink hollow circles) and without PLL (black squares). The PLL-induced step is much higher than the characteristic noise level (0.5 mA ≫ 0.5 μA).

The IVC of the JJ array when the PLL mode is active is shown in Figure 10. The current step induced by the PLL system is approx. 0.5 mA, which is three orders of magnitude higher than the characteristic noise level in the current bias system (≈0.5 μA) and thermal noise. This shows the robust nature of the PLL. The additional steps of smaller size are likely to be caused by the locking at different harmonics and other sidebands. It should be noted that the form of the IVC in spectral measurements in the cryostat is different from that measured in the LHe probe because the PLL system and the JJ array have comparable impedances.

## Conclusion

Summing up, we state that the described oscillator based on a series-connected JJ array embedded into the central electrode of a coplanar line has sufficient output power and spectral ratio in the PLL mode to operate as LO in SIR. Based on the results presented in this work, we conclude the feasibility of tunable LO covering an entire range from 100 to 700 GHz with the only limitation caused by the transmission coefficient of the matching circuit. We also demonstrated that an increase in tunnel current density through the JJs enables frequency generation at higher frequencies. Furthermore, minor corrections to the CPW topology will help to extend the maximum operation frequency further to the high-frequency region.

## Data Availability

Data generated and analyzed during this study is available from the corresponding author upon reasonable request.
